# Spontaneous attribution of underspecified belief of social partners facilitates processing shared information

**DOI:** 10.1038/s41598-022-19569-8

**Published:** 2022-09-23

**Authors:** Andrea Márta Hegedüs, Ildikó Király

**Affiliations:** Cognitive Psychology Department, Eötvös Loránd University, Budapest, 1064 Hungary

**Keywords:** Psychology, Human behaviour

## Abstract

The main question of Theory of Mind research is not only how we represent others’ mental states, but also how these representations influence our first-person interaction with our surrounding environment. A novel theory of belief files proposes that we should think about belief tracking as an online, spontaneous, and effortless mechanism giving rise to structured representations, thus easing the use of beliefs in behavior selection. Beliefs are formed by two different sub mechanisms: (1) opening an empty placeholder belief file, for a particular intentional agent, and (2) filling it up with mental content attributed to the agent. This theory opens the possibility of exploiting theory of mind abilities even in situations when we can attribute only underspecified mental contents to others. The goal of the present study was to provide a proof of concept test: whether spontaneous belief tracking starts effortlessly even when we do not know a partner’s actual belief content. We created an object detection paradigm, where the visual access of a virtual agent to the object to be detected by the participant was manipulated. The agent getting access to the information for processing always preceded the participant getting access to it, resulting in the need of attributing belief without specified content in it. Our results have shown that participants detected the object with a reduced reaction time when the observed agent had visual access to the object’s expected place compared to when the participant watched the same scenario, but the object’s location remained occluded for the observed agent and thus was revealed only for the participant. This suggests that the information processing of humans speeds up when another agent has access to a piece of information as well. Thus, we do track agents’ potential beliefs without knowing its actual content. This study contributes to our understanding of the effect of spontaneous computation of others’ mental states on first-person information processing.

## Introduction

Theory of Mind research has a hard time handling the debate on whether mentalizing is an early emergent, spontaneous process or a late-developing one that requires effortful processing. There is a huge amount of research evidence on making inferences about social partners’ characteristics, based on their appearance or behavior automatically, but it has been a question, whether inferring these partner’s mental content is also an automatic and spontaneous process or not^1^. Cumulative evidence in the field revealed challenging inconsistencies: some studies have found that 3-year-old children fail false belief tasks^2,3^, while other paradigms with different methodologies have shown that infants, namely 18-, 15-, and even 10-month-olds are already capable of understanding false belief situations^4–6^. In addition, research on adults has found evidence that the limited capacity of executive function can be a constraint of the adult theory of mind abilities^7,8^, which indicates that the computation of others’ mental content requires effort. However, in other situations, it was found to operate spontaneously and effortlessly^9–11^ without even being prompted. The challenge is to explain these inconsistencies within a single theory, and we address whether belief attribution as a process is effortful or not.

The theoretical approach provided by Apperly and Butterfill^12^ offers a solution to the above debate by claiming that theory of mind ability consists of two subsystems. One of them computes explicit beliefs in an effortful but flexible way and the other one uses belief-like states in an implicit, effortless but inflexible and limited way. In their view, genuine beliefs are only computed by the more elaborate theory of mind system, and the minimal, early-developing system has signature limits concerning aspectuality. Another viewpoint by Philips and his colleagues^13^ proposes that two different mechanisms of theory of mind ability are knowledge attribution and belief attribution, from which the former one arises both phylogenetically and ontogenetically earlier and can be operated quickly and in a more effortless way. Consequently, their theory also emphasizes that genuine beliefs (i.e. mental content independent of the state of the world, for example, hypotheticals) are handled differently and require mental effort on part of the cognitive system. Indeed, these approaches fail to explain the evidence, that 8-years-old children and adults as well represent others’ level 2 visual perspective spontaneously and effortlessly^14^, despite this ability is supposed to be part of the effortful and flexible system. Second, knowledge attributions are indistinguishable from belief attributions in situations of fast learning: e.g., when they need to locate a target, children cannot distinguish knowledge from mere true beliefs and also they rely on false beliefs to learn about the physical world when they themselves lack perceptual access. It is belief attribution actually that undergirds immediate learning for long-term knowledge^15^.

In brief, the dominant approaches suggest two different cognitive mechanisms to explain belief attribution and the contradictory findings in the literature and support the idea that genuine belief tracking is slow, effortful and emerges later in life.

### A novel solution: theory of belief files

The theory of Ágnes Kovács^9^ suggests a brave alternative, a model which aims to explain how genuine belief attribution could happen spontaneously and fluently in social situations already at an early age. According to this theory^9^, instead of different systems or abilities, different stages of the belief attribution process are proposed. The acts of ‘attributing belief’ and ‘computing belief content’ are not supposed to happen simultaneously. The representational structure which contains the information about others’ knowledge can be described as a *belief file*. Each belief file is assigned permanently to a certain person as a belief holder. To compute an agent’s mental content, the first step is to open a permanent belief file for them. Hence, one does not need to consider an agent’s identity to update their belief content, it is simply enough to update the content of the belief file created earlier. While the files are static and constant, their content, which can be filled at any time point, is not permanent and can change dynamically by any new information complementing or replacing the previous one.

This theory^9^ makes it possible to understand situations where the content of the belief file is unknown, unspecified, or just momentarily irrelevant. Knowledge attribution and belief attribution can be understood as being one process with different stages, as knowledge and belief attribution do not necessarily happen simultaneously. First, I may know that someone can be a source of a possibly relevant piece of information that I am not aware of yet, therefore it may be worth tracking and representing it to effectively interact with them later. For example, during university years, it is always worth tracking who went to a particular class, because at the end of the semester, it is good to know whom to ask about the exam requirements. Second, retrospective inferences about previously unknown contents can also lead us to correct conclusions about the knowledge states of social partners. Imagine going on a hike with a friend and having only one binocular to share. Even if they are the first to use it at a mountain top, and we cannot see the same view simultaneously, after they pass it to us, we can infer that this is the same view they saw before and can have a conversation about nice mountains in the distance.

### Empirical evidence on the impact of belief files on one’s own behavior

The belief files, being complete or incomplete with underspecified content, are sustained in parallel, in a similar format with one’s own representations of the real world^9^. Previous research provided evidence that the contrast between others’ and our own mental representations influences our own behavior^16,17^. Sometimes this contrast is caused by the different focus of attention^11^, by the different visual perspectives^14,18^, or simply by the two agents’ differing experiences about the world^16^. The computation of these incongruent representations happens spontaneously, and it affects our behavior without us being aware of it.

Research results have also shown that our social partners’ perspectives can not only interfere with our own representations, and result in the modulation of behavior, but can also facilitate them^16,17,19^. A study by Freundlieb and his colleagues^17^ has found evidence of the spontaneity of visual perspective-taking and its effect on goal-directed actions in a non-communicative situation. Also, the inhibition of actions can be facilitated by the presence of others^19^. In Frischen and colleagues’ selective reaching task they found that after a location was primed to be inhibited for the co-actor (the co-actor was the first to act in the trial), participants also showed inhibition of reaching towards the same location, and their reaction times increased in the dual person condition. Furthermore, Kovács and her colleagues^16^ have shown that the mere presence of an agent triggers belief computation and the agent’s false belief speeds up the participants’ object detection, despite that the actual belief of the observer was incongruent with the outcome.

These results speak for the interpretation that not only the visual perspective but also the action plan, and the different belief of the social partner is computed spontaneously and effortlessly in the above situations, and these computations had an impact on the actions performed by the observer. Based on the above evidence we claim that belief tracking mechanisms work effortlessly and spontaneously, in accordance with the comprehensive theoretical framework by Kovács^9^.

Importantly, recent research from Kovács^20^ has also shown that already at a very early age, attributing beliefs with ambiguous content primes behavior. In their study, 15-months-old infants participated in an object search task. They observed an agent hiding an object in one of two possible locations, but only the agent could see in which one. At the first hiding phase, the infant could attribute knowledge about the location of the object to the agent but without the concrete value of this variable of the object. After the first hiding phase, the location was revealed (but not the object) and the object was relocated by another experimenter. In the situation when the location of the object was revealed later for the infant, but afterward the agent could not observe the relocation phase, infants were primed to search the object in the location where the agent initially hid the object, and where the agent thought the object was. This means—in the terminology of the Belief File Theory^9^—that it is possible to attribute ambiguous knowledge in early infancy, namely to open a belief file with unspecified content. This idea furthermore suggests that we use our incomplete knowledge about others’ mental states to plan our actions the most effective way.

In light of the above theory, however, the question emerges whether the process of belief attribution and monitoring itself or the successful filling in of the belief content serves as the basis for the influence on one’s own behavior. Kovács’s theory^9^ offers a testable hypothesis for this question since the mere presence of our social partner should trigger the opening of a belief file, and only afterward—when we get access to the particular information -, one may fill the file with belief content. So it is proposed that the influence of this—supposedly spontaneous mentalization—process comes into play already when one is expected to open a belief file, and not necessarily when it is filled with content. A related, alternatively formed question is whether maintaining an incomplete representation affects behavior the same way as beliefs with known content do? Based on the above prediction arising from Kovács’s theory, we anticipate that attributing underspecified beliefs influences behavior similarly to specified beliefs. Our goal was to provide experimental evidence which investigates spontaneous attribution of unspecified beliefs and identifies the opening of a belief file, even without specified content, in adults.

### Methodological considerations of the study

**Figure 1:**
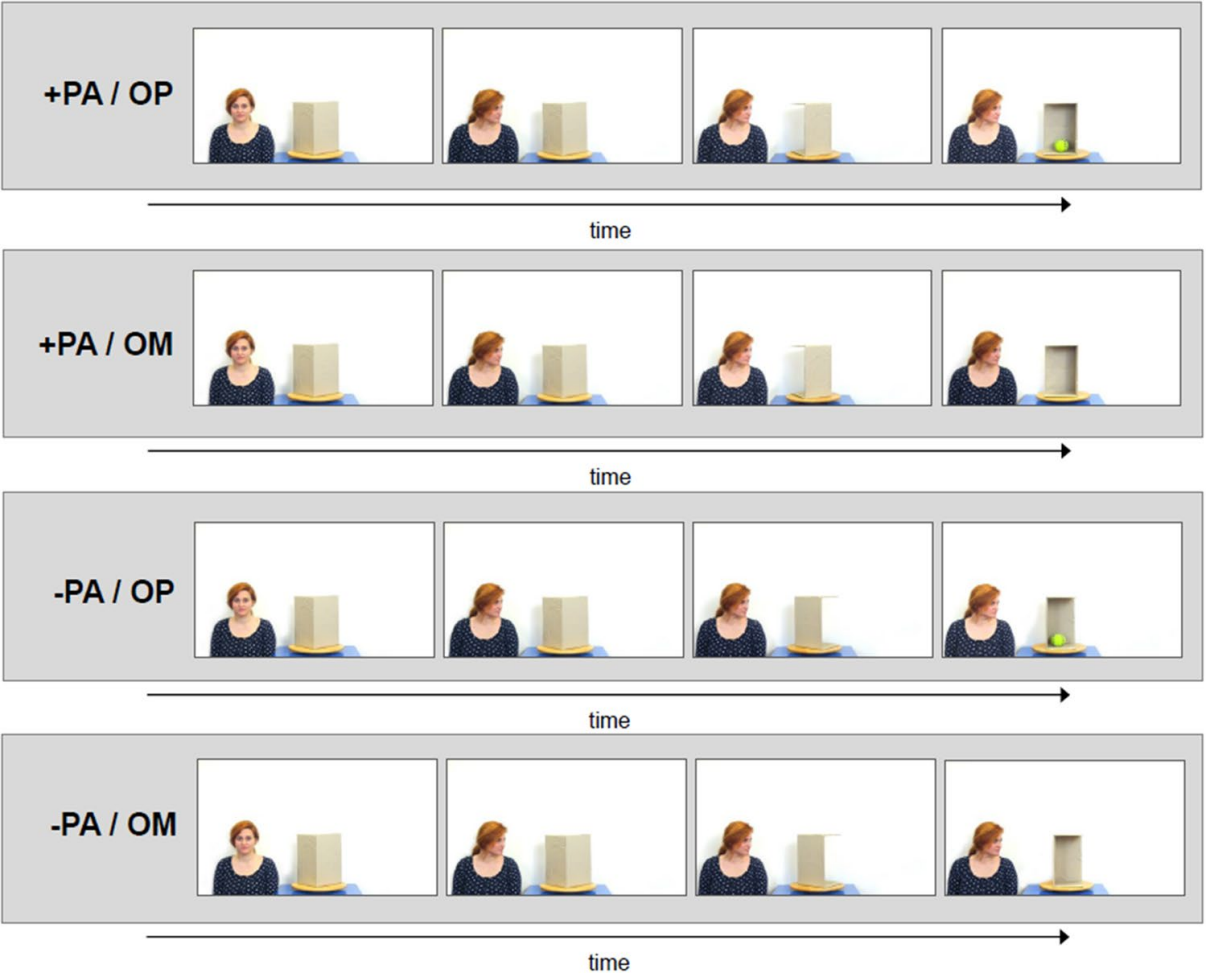
The experimental conditions of the study. In the ambiguous content in the perceived access (+ PA) condition, the box rotates toward the agent, while in the perceived non-access (−PA) condition the box rotates the other way around. At the end of the events, either an object was present (OP) or not (OM). Person in the stimulus has signed an informed consent form of publication of identifying images in an open-access publication.

We created a situation to model this scenario with an object detection task and employed a 2 × 2 design manipulating the perceived access of a virtual observer to the object’s potential location and whether a particular object was present (Fig. 1). In the task, a virtual agent was present on the screen and we manipulated whether the participants saw the agent had visual access to the potential location of the object (Perceived Access (+ PA) condition) or that the participants saw that the agent had no access to the location (Perceived Non-access (−PA) condition). Otherwise, the stimuli remain visually equivalent for the participant across different conditions. The other factor in the experimental design was whether the object to be detected was present (Object Present (OP) condition) or not (Object Missing (OM) condition). We expect that the perceived information access of the agent will facilitate the response time of participants.

There are three essential points concerning this scenario. First, we manipulated not only one feature of a belief content (e.g. location of the object or its color) but the availability of the content itself. In Kovács et al.’s study^19^ the location of the object was manipulated, which means the belief content was ambiguous only regarding the object’s location but not regarding the content per se. As long as we share any visual field with a social partner—therefore we know at least the context of what she has visual access to—we cannot keep the belief file attributed to her empty, so the effect of content can be investigated on different levels of specificity. Then, to minimize the shared information content, in the present study the presence of the attended object was underspecified and not certain features of it.

Second, previous studies have shown that gaze cueing has a facilitating effect on information processing, which means that we process stimuli faster and more efficiently when someone else (or just a pair of eyes) is looking at its particular location^21,22^. This effect is still not fully explained, although according to an evidence-based opinion, gaze cueing facilitates information processing by orienting attention toward its target^19^. In the present paradigm, the attention orienting effect of a gaze shift was controlled: looking behaviour was present in both conditions, but only led to access to the object location in + PA condition. With the help of this manipulation, we should predict that any difference between the two conditions is caused by the difference in the perceived access of the observer to the location where the information occurred, not by the mere fact that the location was looked at by the observer.

This assumption leads to our third consideration, namely the possibility and consequences of visual perspective-taking (VPT), a skill that is considered one of the very first sub-processes to develop in the theory of mind ability, phylogenetically and ontogenetically as well^23–26^. In some cases we cannot base our inferences about others’ mental content only and exclusively on their assumed visual access. One might also take into account the selective nature of their attention, thus consider not only what they can see, but what they prefer to see, or what they attend to^14^. The below paradigm was designed to ensure that participants are aware that the expected location of the object is in the focus of the agent’s attention—since the agent takes an action and turns toward the box in every trial. The aim of the study was twofold. First, one of the main goals was to investigate whether attributing beliefs with underspecified content can lead to a facilitation effect on one’s own actions, as we saw in the research described above. The second purpose was to create a social situation as simple as possible, with manipulating only and exclusively the visual access of an agent in a very simple object detection task. We assume that attributing mental states without content will also influence the goal-directed actions of participants, and the attribution of potential knowledge that the participants are not yet aware of will also decrease reaction time in this task.

## Method

### Participants

Data were collected from 53 adults—mainly university students (42 females, 11 males, M_age_ = 24.07 SD = 6.46), participation was rewarded by course credits. The experiment was carried out in accordance with relevant guidelines and regulations, and it was approved by Ethics Committee of Faculty of Education and Psychology of Eötvös Loránd University which functions in accordance with the Ethical Code for the Subjects of Eötvös Loránd University. Before starting the experiment, the participants signed an informed consent form.

## Materials and procedure

The stimuli were presented on a 1366 × 768-pixel screen using OpenSesame (version 2.8.3). Responses were recorded by a button press on a response box (Cedrus RB-530) measuring their reaction time.

Participants were seated in front of a monitor with the response box located in front of it on a table. Participants were instructed to place their two index fingers on the two buttons of the response box, a red one on the left side and a green one on the right side.

In the trials of the experiment, participants were watching a sequence of pictures. In the beginning, they were instructed to decide whether there is an object inside the rotating box or not as soon as they can see its content by button press. They were instructed to place their index fingers on the red and green buttons of the response box and press the red button in case the object was absent and the green button if the object was present. In the pictures, a box with its two sides removed was rotating towards them in 4 phases (see in Fig. 1) with its opened side back, so the content of the box was revealed only in the last picture. In each test trial, a female agent was standing on the left side of the box. In the first phase, she looks into the camera (so she cannot see the box, which stands there with its open side back). In the second phase, she shifts her gaze toward the box, but cannot see what is inside the box, since it is obscured for her by the box’s one side. In the third phase, the box turns with its open side toward her—that is the phase when she got access to the content of the box. In the third phase, for the participant, the content is still obscured by the box’s one side. In the fourth phase, the agent remains still, but the box turns with its opened side toward the participant. The first three stages were presented for 1000 ms, the last stage was presented for 1500 ms. Based on the results of pilot studies, response time limit was set to 1500 ms. If the participants did not press any button within this time frame, the task moved on to the next trial. The critical manipulation that differentiated the conditions was the direction of the rotation of the box. In the Perceived Access (+ PA) condition, the box rotates toward the agent, so she has access to the content of the box sooner than the observer and detects if something is in the box already in the third phase. In the Perceived Non-Access (−PA) condition the box rotates the other way around, thus the model cannot see the content of the box during the entire scenario.

The experiment consisted of five main blocks: one practice block and four test blocks. In the practice block (8 trials) only the rotating box (without an agent next to it) was presented with two kinds of trials varied randomly (Object Missing (OM) /Object Present (OP)). We used a 2 × 2 design in the test phase, manipulating the direction of rotation and therefore the visual access of the agent (+ PA/−PA) and the presence of the object (OM/OP). In each of the test blocks, 46 trials were presented and there was a 1-min long break after each of them. The task altogether was approximately 25 min long.

We predict the main effect of the + PA/−PA condition on the reaction time, namely that reaction time will be decreased in + PA condition compared to −PA condition. We also predict the main effect of the + PA/−PA condition on accuracy, as facilitating effect of the observer’s perceived access will lead to a better information processing, this way to a bigger hit rate in the + PA condition.

## Results

### Reaction time

We analyzed the reaction times for correct responses only. We used a fixed time limit (1500 ms) for the responses in order to keep the participants focused and motivated. Indeed, with this response constraint, only 1.6% of all datapoints were above the mean with three times the standard deviation. We decided to keep those datapoints in order to maximize the number of valid data in our sample.

The normality of reaction time data was assessed. Kolmogorov–Smirnov test indicated that results were normally distributed in three of our 2 × 2 conditions (+ PA/OM: D(53) = 0.091, p = 0.2; + PA/OP: D(53) = 0.095, p = 0.2; −PA/OM: D(53) = 0.106, p = 0.2). In −PA/OP condition Kolmogorov–Smirnov test was significant (D(53) = 0.122, p = 0.047). As only one condition was not normally distributed, it was suitable for parametric comparison between conditions, although to ensure the reliability of our analysis we ran non-parametric tests as well.

### ANOVA

We performed mixed ANOVAs on the mean of reaction times. As 79% of the participants were female, first, we added gender as a between subject factor, but it had no significant main effect on reaction time (t(52) = 0.023; p = 0.881).

**Figure 2:**
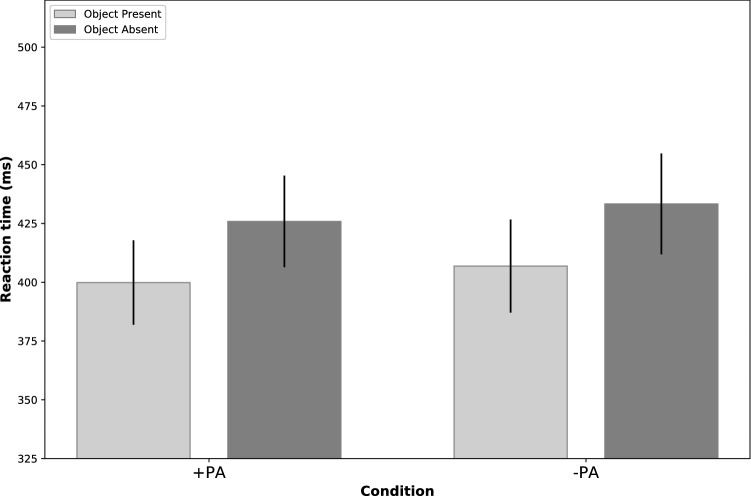
Average reaction times. On the X-axis there are the two conditions: + PA, when the observer’s perceived having access to the object’s potential location, and −PA where the observer is perceived having no access to it. Y-axis shows reaction time in milliseconds. Error bars indicate 95% CI in each condition. Post hoc analysis revealed that reaction times were significantly lower (in the + PA condition (t(52) = −2.833, p = 0.07, d = −0.389) and when the object was present (t(52) = −3.137, p < 0.001, d = 0.964). This pattern of results suggests that the perceived access of an observer to a particular information that participants processed had a facilitating effect on the speed of object detection.

We performed a 2 (Object Presence: Object Present (OP)/Object Missing (OM)) × 2 (Access: + PA/−PA) mixed ANOVA on the mean of reaction times (Fig. 2). We found a significant main effect of both of the Object condition (F = 49.306, p < 0.001, η^2^p = 0.487) and the Access condition (F = 8.026, p = 0.007, η^2^p = 0.134) with shorter RT in + PA, but the interaction between these two factors was not significant (F = 0.014, p = 0.907). In the post hoc analysis, we conducted pairwise comparisons of the two test conditions, and a significant difference was found between the two levels of the object condition (M_OM_ = 429.0, SD_OM_ = 75.82,_,_ M_OP_ = 403.1, SD_OP_ = 69.7, t(52) = −3.137, p < 0.001, d = 0.964), which revealed that participants were quicker when the object was present. We also found a significant difference between the two levels of the Perceived Access condition, the RT was significantly lower in the, + PA condition (M_+PA_ = 412.7, SD_+PA_ = 70.4, _,_ M_−PA_ = 419.5, SD_−PA_ = 77.2, t(52) = −2.833, p = 0.07, d = −0.389).

### Non-parametric analysis

We used the non-parametric analysis of Generalized Linear Mixed Models (GLMM) to test for the differences in reaction times across conditions. Gender as a variable was included in the initial model, but was later removed as it was not significant. “Participant” was added as a factor and “Perceived Access” and “Object Presence” were included as repeated measures in the final model. The Perceived Access condition had a significant main effect on the reaction time of the participants, with shorter reaction times in the + PA condition as opposed to the −PA condition (F = 6.491, p = 0.012). In addition to this, the Object Presence condition had also a significant main effect, with longer reaction times in the OM condition as opposed to the OP condition (F = 25.867, p < 0.001). No significant interaction was revealed.

### Accuracy

#### ANOVA

We also performed mixed ANOVAs on the hit rates. In the same way as in reaction time data, first, we added gender as a between subject factor, but it had no significant main effect on reaction time (t(52) = 0.206; p = 0.652).

**Figure 3:**
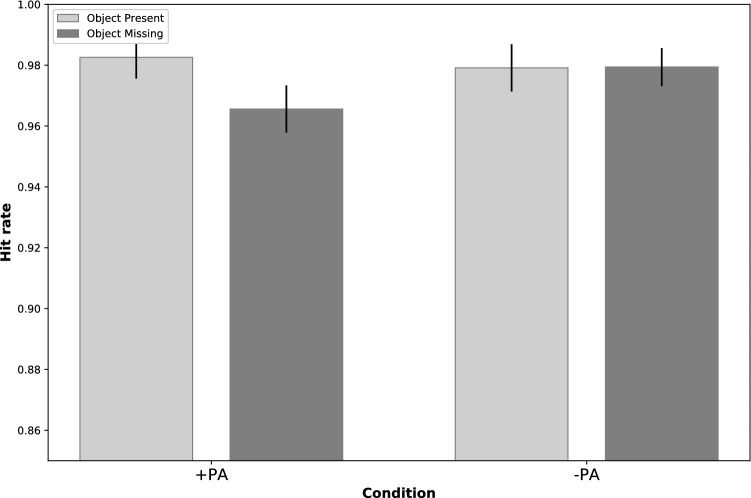
Hit rate results by conditions. On the X-axis there are the two conditions: + PA, when the observer’s perceived to have access to the object’s potential location t, and −PA where the observer is perceived as having no access to it. The Y-axis shows hit rates. Error bars indicate 95% CI in each condition.

We performed a 2 (Object Presence: OM/OP) × 2 (Perceived Access: + PA/−PA) mixed ANOVA on the hit rates of the different trials (Fig. 3). In the experiment, the overall average hit rate was 98.02%. The ANOVA revealed the significant main effect of the Object condition (F = 9.84, p = 0.003, η^2^p = 0.159) with a higher hit rate in OP condition and a significant interaction between the two conditions (F = 14.916, p = 0.001, η^2^p = 0.223), wherein + PA condition there was a higher hit rate in OP (M_OP_ = 0.984, SD_OP_ = 0.02) than OM (M_OM_ = 0.997, SD_OM_ = 0.028), meanwhile, the main effect of the Perceived Access condition was only marginally significant (F = 3.813, p = 0.056) with higher hit rate in PA− (M_−PA_ = 0.983, SD_−PA_ = 0.023) than PA + (M_+PA_ = 0.977, SD_+PA_ = 0.028) . The post hoc analysis showed that there was a significant difference in accuracy between the + PA/OM (M = 0.969, SD = 0.030) and the + PA/OP (M = 0.986, SD = 0.022) conditions (t(52) = –4.247, p < 0.001, d = −0.583), meaning that in those trials, when the agent was aware of the content of the box, hit rate was higher in the OP trials than in the OM trials. Also a significant difference between the + PA/OM (M = 0.969, SD = 0.03) and -PA/OM conditions (M = 0.984, SD = 0.023) (t(52) = 3.341, p = 0.002, d = −0.459) was revealed, which means the participants’ hit rate was lower in those OM trials when the agent was aware of the content of the box.

Post hoc analysis revealed that hit rates were specifically lower (t(52) = –4.247, p < 0.001, d = −0.583) in the + PA/OM condition than in the + PA/OP condition. This pattern of results reflects that the decision that there is no object on the scene when the observer is perceived to have access to the location e is more difficult than detecting the presence of an object in the same situation.

### Non-parametric analysis

With accuracy measures, we also used the non-parametric analysis of Generalized Linear Mixed Models (GLMM) to test for the differences in response patterns across conditions. Here again, “Participant” was added as a factor and “Perceived Access” and “Object presence” were included as repeated measures in the final model. The Perceived Access condition had a significant main effect on the hit rate of the participants, with better performance in the + PA condition as opposed to the −PA condition (F = 0.016; p = 0.022). In addition to this, the Object Presence condition had also a significant main effect, with more errors in the object missing condition as opposed to the Object Present condition (F = 0.017; p = 0.005). The interaction of these factors, the Perceived Access and Object Presence factors was also significant, (F = −0.019, p < 0.001) This pattern of data is in line with the results revealed by the ANOVA.

## Discussion

The main goal of our study was to investigate whether in a minimal social situation humans take into consideration the perception-based, potential knowledge of a partner and whether one’s own behavior is affected by the underspecified mental state of the partner or not.

Our results revealed that participants detected the object on the screen faster and with less error when another observer could have had the possibility to see it before them. We would like to acknowledge, indeed, that our method measures spontaneous inferences in the form of delay in reaction times and behavioral mistakes, and therefore we can provide only indirect evidence on the phenomenon in question. Importantly, the crucial difference between the conditions in this study was only that the observed partner could or could not have visual access to the possible location of an object before the observer.

Based on the theory of Kovács^9^ we argued that when we are aware of a social partner having access to possibly relevant information, the knowledge attribution process starts—spontaneously and effortlessly—with opening a placeholder, a so-called belief file for the agent, even without any or with ambiguous content. In this perspective, looking behavior induces the attribution of intentional attention on part of the protagonist (see for a review^27^).

An alternative possibility would be, however, that gaze shift or looking modulates only the amount of attention paid to a location. Indeed, in both conditions, the agents shifted their gaze and looked towards the anticipated location. Therefore, the difference found between + PA and −PA conditions could be interpreted as an effect emerging from the interpretation of the relation between the looking behavior and the perceived constraints of it, and not simply as the modulation of attention towards the gazed-at location^28^. In fact, there is a further alternative: observing looking towards a location together with monitoring the access space of the looking behavior could induce an expectation of an object being there. We need to acknowledge, however, that this processing is inherently relational: assumes that seeing has its specific constraints, (e.g. one cannot see through obstacles) and requires a minimal understanding of the intentional nature of the process of seeing (one perceives only what is in the accessible space). This leaner interpretation, however, could interpret as well the findings that when an object was missing, the reaction times were higher in the + PA than in the −PA condition, without belief attribution. Still, this interpretation also recruits intentional attention attribution.

An intriguing pattern in the results is that the reaction time was longer in cases when the object was absent. It is still not completely clear how we represent negation, the non-existence or absence of something^29,30^, but our cognitive system seems to prefer representing unequivocal but not ambiguous contents^31^. This is in accordance with the fact that representing something’s nonexistence is a challenging situation for our cognitive system, confirmed by the empirical evidence that understanding negation in comprehension tasks is found to increase response latencies^32^. This difficulty explains the main effect of the Object Presence condition since overall reaction time was longer when the participant had to process the absence of the object and represent it as missing. This main effect emerged independently of social influences, and we assume that this phenomenon is rooted in first-person representation formation. However, accuracy results seem to reflect the social aspects of this process as well. Importantly, hit rates decreased in + PA/OM condition, where the agent had access to the information that the object is not present. We suppose that the interaction found reflects the difficulty of attributing a content to an agent which contains information about the absence of an object, in other words, the negation of the existence of that object (not only for the self but for the agent as well). Accuracy in this very easy object detection task seems to be sensitive enough to reveal the readiness to attribute belief files with concrete content in them to those social partners who potentially have relevant knowledge. Thus, accuracy results show a proof of concept, since the difference between critical conditions (+ PA / −PA) reveals how different those situations are when we attribute knowledge without content compared to those, where we do not even start the mental state attribution process.

Consequently, the implicit effect of our social partners’ presence on our cognitive mechanisms prevails even when our visual fields are not shared completely, therefore the content of the partner’s belief is hidden from ourselves. Based on these results we conclude that the partner’s perceived access to a potential—underspecified—information, had a facilitatory impact on one’s behavior, just as it has been shown by previous research ^14,16–19^.

In order to test the theoretical possibility, whether underspecified information inferred from potential access could influence one’s own behavior, the simplest possible experiment was designed. Indeed, the fact that this is a single experiment study with simple controls in it, represents a significant limitation. In the future it is necessary to supplement this dataset with different controls, for example with manipulating the observer’s characteristics, the object’s characteristics, or changing the way of manipulating visual access of the observers.

## Summary

The main findings confirm that mentalization and belief tracking mechanisms work easily, effortlessly, and spontaneously without prompt. Furthermore, results provided by this experimental design could prove that this process is being conducted even in an implicitly and minimally social situation, where we cannot even attribute actual beliefs to a social partner, our only available information being their *potential knowledge* about a potentially relevant piece of information. These findings can raise questions for the field about the nature of the theory of mind ability. These findings interpreted in Kovács’s theoretical framework^9^ support those theories that question the dominance of dual process and dual system approaches^12,13^, in respect to that intention reading and attention attribution requires effort, and may motivate further research to move forward in the revision of these considerations and to start approaching belief tracking as an online monitoring process that can start effortlessly without any prompt.

## Supplementary Information

Supplementary Information.

## Data Availability

All data generated or analyzed during this study are included in this published article and its supplementary information files.
